# The association between heat stroke and subsequent cardiovascular diseases

**DOI:** 10.1371/journal.pone.0211386

**Published:** 2019-02-13

**Authors:** Jen-Chun Wang, Wu-Chien Chien, Pauling Chu, Chi-Hsiang Chung, Chih-Yuan Lin, Shih-Hung Tsai

**Affiliations:** 1 Department of Emergency Medicine, Tri-Service General Hospital, National Defense Medical Center, Taipei, Taiwan; 2 Institute of Clinical Medicine, National Yang-Ming University, Taipei, Taiwan; 3 Department of Medical Research, Tri-Service General Hospital, National Defense Medical Center, Taipei, Taiwan; 4 School of Public Health, National Defense Medical Center, Taipei, Taiwan; 5 Taiwanese Injury Prevention and Safety Promotion Association, Taipei, Taiwan; 6 Division of Nephrology, Department of Medicine, Tri-Service General Hospital, National Defense Medical Center, Taipei, Taiwan; 7 Department of Surgery, Division of Cardiovascular Surgery, Tri-Service General Hospital, National Defense Medical Center, Taipei, Taiwan; 8 Department of Physiology and Biophysics, Graduate Institute of Physiology, National Defense Medical Center, Taipei, Taiwan; University of Mississippi Medical Center, UNITED STATES

## Abstract

**Background:**

Recent studies have indicated that several critical illnesses are associated with an increased risk of cardiovascular diseases (CVDs). Nonetheless, studies of the association between heat-related illnesses (HRIs) and subsequent CVDs are still limited. We sought to evaluate whether heat stroke (HS) was associated with an increased CVD incidence.

**Methods:**

The data from the nationwide, population-based, retrospective, cohort study described herein were obtained from the National Health Insurance Research Database in Taiwan. The outcome evaluated in this study was the cumulative incidence of CVDs, which was compared between patients with HS, patients with other HRIs and a control group during a 14-year follow-up period.

**Results:**

Our analyses included 150 HS cases, 150 patients with other HRIs and 150 patients without HRIs. The HS patients had a significantly higher incidence of developing CVDs than the other HRI and control patients (32.67% vs. 23.33% vs. 16.67%, p = 0.005). Patients with HS had an increased incidence of acute myocardial infarction (AMI) compared with that of the controls (6% vs. 2.67%, p = 0.042) and an increased incidence of acute ischemic stroke (AIS) compared with those of the other HRI and control patients (12% vs. 6% vs. 4.67%, p = 0.038). An increased risk of chronic kidney disease (CKD) was also found in the patients with HS and other HRIs compared to that in the controls (17.33% vs. 14.67% vs. 6.67%, p = 0.016).

**Conclusion:**

Prior HS was associated with an increased incidence of CVDs, particularly AMI and AIS, and an increased incidence of CKD.

## Introduction

Heat-related illnesses (HRIs) affect a large number of people every year and are becoming an increasingly common cause of health issues, as climate change is resulting in rising global temperatures [1]. Patients with HRIs present to the emergency department frequently, and the visit rates are correlated with temperature anomalies [2]. Severe HRIs can cause multiple organ failure, rhabdomyolysis and coma with acute and even permanent damage to the vital organs [3–6]. Heat stroke (HS) is the most severe HRI and has been defined as a patient with profound central nervous system abnormalities and severe hyperthermia (core temperature typically but not always > 40°C) [7]. HS is associated with high mortality, and survivors may retain chronic neurological sequelae [8].

Factors that predispose individuals to HRIs include a pre-existing illness, cardiovascular diseases (CVDs), a prior HRI, extremely high body mass index (BMI), certain medications, tobacco use, a poor fitness level and the sickle cell trait [7,9,10]. A previous study indicated that HRIs were associated with increased mortality due to CVDs during hospitalization [11]. Recent studies also indicated that several critical illnesses were associated with an increased risk of CVDs, such as acute ischemic stroke (AIS) and acute myocardial infarction (AMI). Nonetheless, studies of the association between HRIs and subsequent CVDs are still limited.

Therefore, we sought to use cohorts from the National Health Insurance Research Database (NHIRD) and evaluate whether HS was associated with an increased incidence of CVDs.

## Methods

### Data source

The NHIRD contains outpatient and inpatient claims for all beneficiaries enrolled in Taiwan’s mandatory National Health Insurance (NHI) program and includes records for more than 99% of the Taiwanese population (more than 23 million people). The NHIRD contains patient identification numbers, birthdays, sexes, dates of admission and discharge, ICD-9-CM (International Classification of Diseases, 9th Revision, Clinical Modification) diagnostic codes (up to five each) and outcomes. In this study, we used data from the Longitudinal Health Insurance Database (LHID), which is a subset database selected randomly from the NHIRD. The LHID contains information on medical service utilization for approximately one million beneficiaries who represent approximately 5% of the Taiwanese population. A random number generator was used to select 1,000,000 sample patients from a population containing 22,717,053 persons. The random number generation was executed by Oracle’s internal random number generator. In this study, information was extracted from the NHIRD between 2000 and 2013. Previous studies have validated the accuracy of the diagnoses of major diseases in the NHIRD, including acute coronary syndrome and stroke [12]. This study was approved by the Institutional Review Board of Tri-Service General Hospital at the National Defense Medical Center in Taipei, Taiwan (TSGH IRB No. 2-105-05-082). The requirement for informed consent from each patient was waived.

### Sampled patients

This study included the study cohort and a comparison cohort. Using the LHID dataset, patients aged ≥ 20 years who were newly diagnosed with HS (ICD-9-CM 992) or another HRI (ICD-9-CM 992.1 through 992.9 (heat syncope, heat cramps, heat exhaustion, heat fatigue, heat edema and other unspecified effects)) and were followed up between 2000 and 2013 were enrolled. We excluded patients who had heat stroke before the index date, cardiovascular events before tracking, an age less than 20 years old, an unknown sex and a medical history of digoxin and warfarin use. We also excluded patients with a follow-up duration of less than 6 months. The date of the HRI diagnosis was used as the index date. The control patients were selected from individuals in the LHID who had no history of HS or HRI. The patient and control cohorts were selected by 1:1:1 matching according to the baseline variables age, sex, comorbidities, including hypertension (ICD-9-CM 401–405), diabetes mellitus (ICD-9-CM 250), hyperlipidemia (ICD-9-CM 272.0–272.4), chronic obstructive pulmonary disease (COPD, ICD-9-CM 490–496), alcoholism (ICD-9-CM 303), obesity (ICD-9-CM 278), cancer (ICD-9-CM 140–208) and chronic kidney disease (CKD, ICD-9-CM 580–589) and annual medical follow-ups. The medication history, including the use of β-blockers, calcium channel blockers (CCBs), angiotensin-converting enzyme inhibitor (ACEi), angiotensin receptor blocker (ARB), diuretics, statins and steroids, was also obtained.

### Outcome measures

The outcome evaluated in this study was the cumulative incidence of CVDs, which was compared between patients with HS, patient with other HRIs and the control group during the 14-year follow-up period.

### Statistical analysis

The clinical characteristics of the patients enrolled in the study are expressed in numerical form. Categorical variables, which are presented as percentages, were compared using Fisher’s exact test and the Chi-square test. One-way AVONA was used for comparisons among groups. The Chi-square and Fisher’s exact test were applied as post hoc tests for categorical variables, and the Bonferroni test was applied for continuous variables. Continuous variables were presented as the mean and standard deviation and were compared using t-tests. Propensity matching analysis was performed with the logistic regression model. The potential confounders were the index year, sex, age, comorbidities and medications. The match tolerance was 0.15 based on the nearest neighbor method. The study-comparison cohort matching ratio was 1-fold. The primary goal of the study was to determine whether patients with HS were associated with the development of CVDs. Associations between time-to-event outcomes (prognoses) and clinical characteristics were examined using Kaplan-Meier survival analysis and multivariate Cox regression analysis. The results are presented as adjusted hazard ratios (HRs) with corresponding 95% confidence intervals (CIs). Statistical significance was indicated at p < 0.05. All data analyses were conducted using SPSS software, version 22 (SPSS Inc., Chicago, IL, USA).

## Results

**Fig 1:**
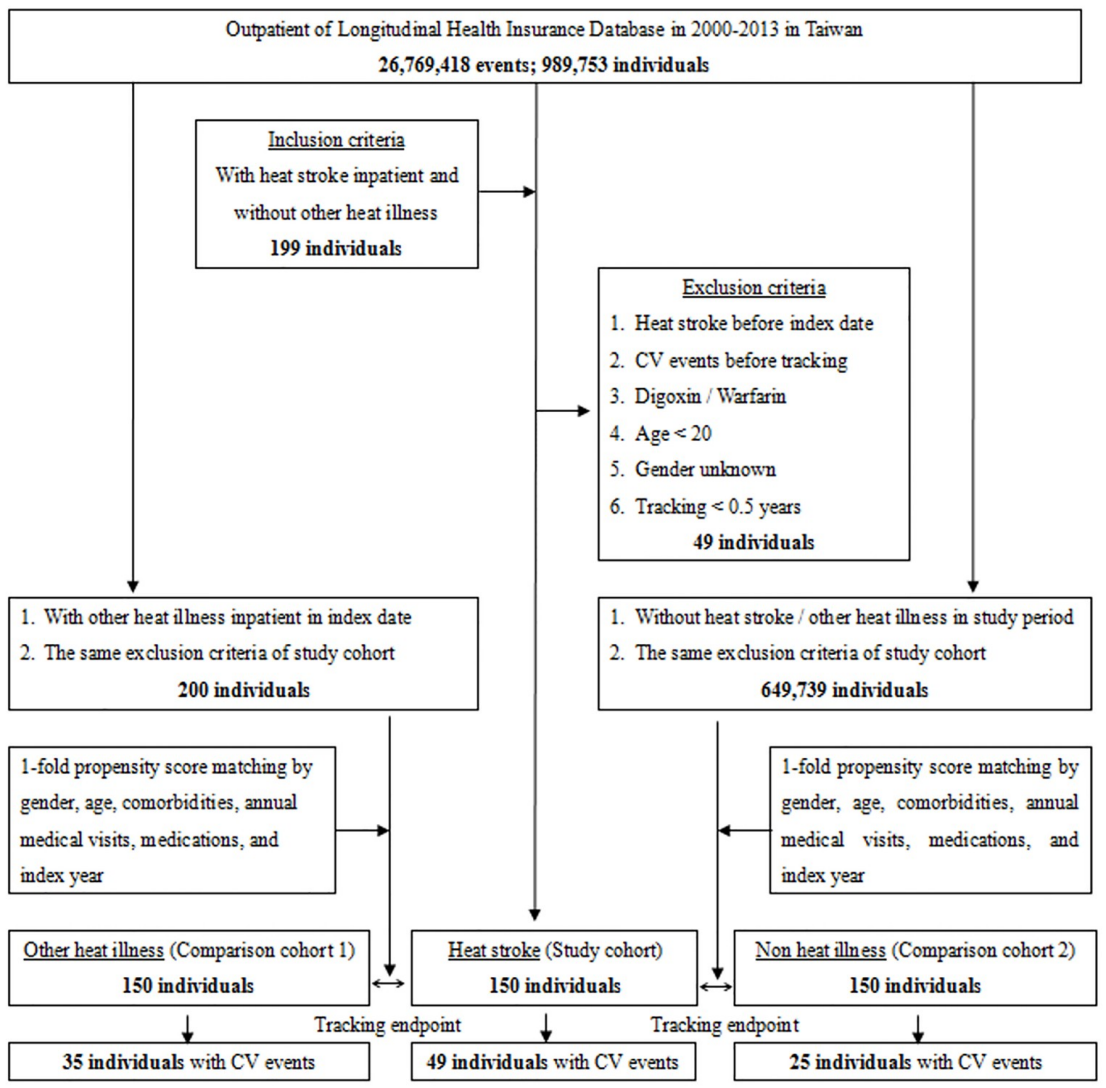
Patient selection flowchart. CV events = cardiovascular events.

Fig 1 presents a flow diagram of patient enrollment in this study. Among a total of 989,753 patients in the LHID from the NHIRD, 150 individuals with heat stroke were identified. Another 150 sex- and age-matched individuals were designated to each of the other HRI and control groups. Table 1 shows no significant differences in sex, age, comorbidities, medications and the number of medical follow-ups among the groups at baseline after matching.

**Table 1:** Characteristics of the study participants at baseline.

Heat-related illness	Heat stroke (n = 150)		Other heat-related illnesses (n = 150)		Non-heat-related illnesses (n = 150)		p	Post hoc test
Characteristics	N	%	N	%	N	%		
**Sex**							0.999	
Male	135	90.00	135	90.00	135	90.00		
Female	15	10.00	15	10.00	15	10.00		
**Age (years)**	44.62 ± 20.35		45.88 ± 19.72		46.67 ± 17.89		0.487	
**Comorbidities**								
HTN	14	9.33	13	8.67	15	10.00	0.924	
DM	14	9.33	16	10.67	14	9.33	0.904	
Hyperlipidemia	5	3.33	4	2.67	4	2.67	0.924	
COPD	3	2.00	4	2.67	3	2.00	0.903	
Cancer	3	2.00	5	3.33	4	2.67	0.773	
CKD	20	13.33	18	12.00	22	14.67	0.794	
**Medications**								
β-blocker	12	8.00	11	7.33	12	8.00	0.969	
CCB	8	5.33	7	4.67	9	6.00	0.876	
ACEI	10	6.67	9	6.00	11	7.33	0.898	
ARB	9	6.00	8	5.33	8	5.33	0.959	
Diuretic	6	4.00	6	4.00	6	4.00	0.999	
Statin	6	4.00	7	4.67	5	3.33	0.841	
Steroid	2	1.33	2	1.33	1	0.67	0.817	

HTN = hypertension, DM = diabetes mellitus, COPD = chronic obstructive pulmonary disease, CKD = chronic kidney disease, CCB = calcium channel blocker, ACEIs = angiotensin converting enzyme inhibitors, ARBs = angiotensin receptor blockers

p: Chi-square/Fisher’s exact test for categorical variables and one-way ANOVA for continuous variables

Post hoc test: Chi-square/Fisher’s exact test for categorical variables and Bonferroni for continuous variables

**Fig 2:**
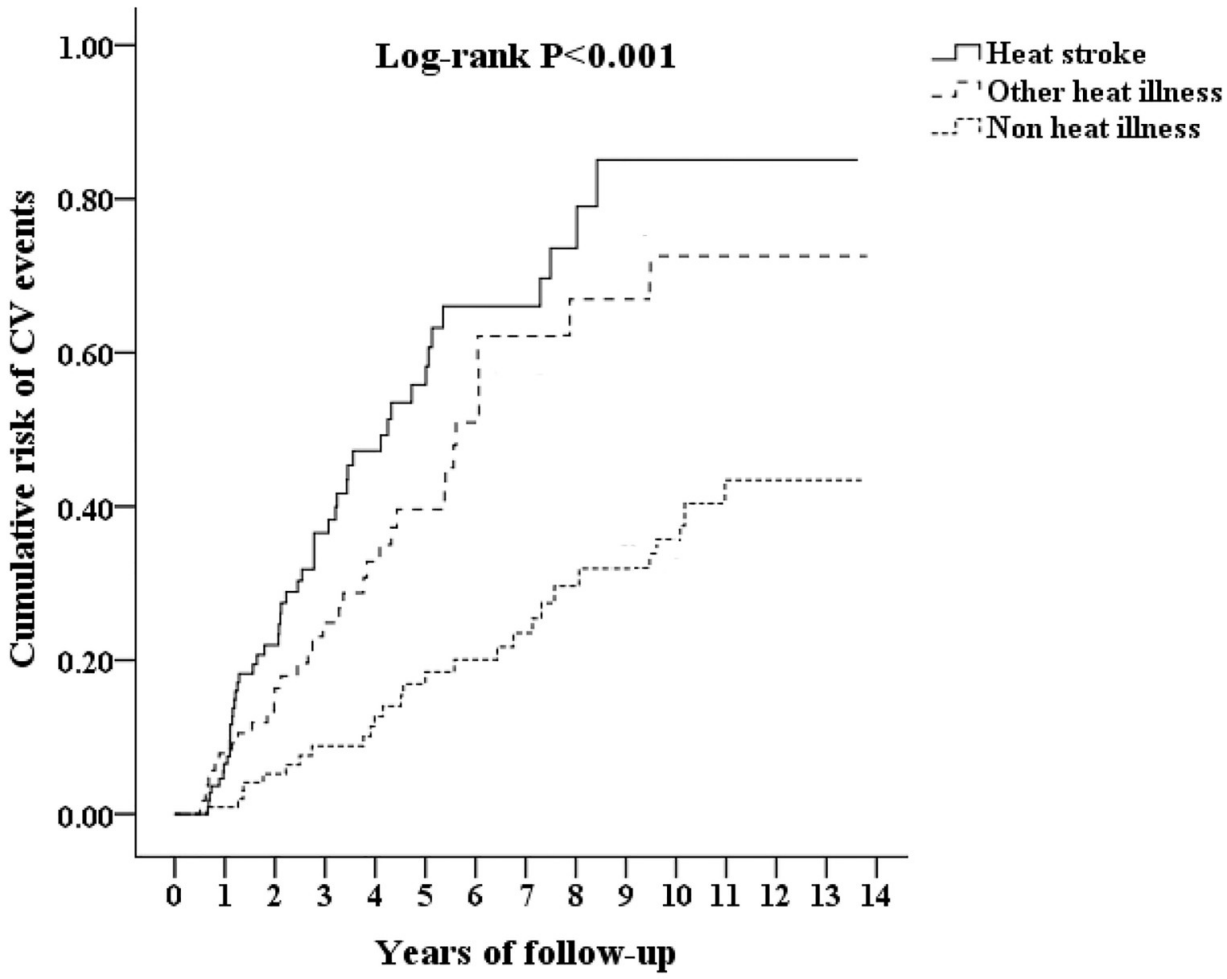
Comparison of the cumulative incidence of cardiovascular diseases among patients with heat stroke, other heat-related illnesses and Non-heat-related illnesses. CV events = cardiovascular events. * p values < 0.05 were considered statistically significant.

Kaplan-Meier analysis for the cumulative incidence of CVDs in the HS, other HRI and control groups is shown in Fig 2. Patients with HS had a significantly increased cumulative incidence of CVDs compared to those of the patients with other HRIs and the controls (log rank test < 0.001) starting the third year after the index event.

Table 2 demonstrates the incidence of CVDs during the fourteen-year follow-up period. At the end of follow-up, patients with HS had significantly higher composed incidences of CVDs than those with other HRIs and the controls (32.67% vs. 23.33% vs. 16.67%, p = 0.005). In the post hoc analysis, patients with HS had an increased incidence of AMI compared with that of the controls (6% vs. 1.33%, p = 0.042). Patients with HS had an increased incidence of AIS compared with those of the patients with other HRIs and the controls (12% vs. 6% vs. 4.67%, p = 0.038). Additionally, an increased risk of CKD was found in the patients with HS and other HRIs compared to that of the controls (17.33% vs. 14.67% vs. 6.67%, p = 0.016). Table 3 shows the Cox regression analysis. Patients with HS exhibited a significantly increased incidence of CVD development compared to those of the patients with other HRIs (adjusted HR = 1.485, 95% CI = 1.019–2.367, p = 0.03) and the controls (adjusted HR = 3.899, 95% CI = 2.275–6.942, p < 0.001). Patients with HS exhibited a significantly increased risk of developing AMI compared to those of the patients with other HRIs (adjusted HR = 7.426, 95% CI = 1.194–45.785, p = 0.009) and the controls (adjusted HR = 2.711, 95% CI = 1.849–13.001, p < 0.001). Patients with HS also exhibited a significantly increased risk of developing AIS compared to those of the patients with other HRIs (adjusted HR = 2.399, 95% CI = 1.275–5.899, p < 0.001) and the controls (adjusted HR = 5.498, 95% CI = 1.306–18.204, p < 0.001). In addition, patients with HS had an increased incidence of IHD (adjusted HR = 3.124, 95% CI = 1.266–7.973, p = 0.003) and AF (adjusted HR = 14.978, 95% CI = 1.785–129.782, p < 0.001).

**Table 2:** Cardiovascular events and characteristics during the 14-Year follow-up period.

Heat-related illness	Heat stroke (n = 150)		Other heat-related illnesses (n = 150)		Non-heat-related illnesses (n = 150)		p	Post hoc test
Characteristics	N	%	N	%	N	%		
**Age (years)**	47.46 ± 20.35		48.29 ± 20.08		50.01 ± 19.74		0.301	
**Annual medical visits**	2.40 ± 3.75		2.34 ± 3.89		3.15 ± 4.90		0.222	
**CV events** (overall)	49	32.67	35	23.33	25	16.67	0.005 **	#, §, †
CAD	24	16.00	21	14.00	10	6.67	0.034 *	§, †
AMI	9	6.00	3	2.00	2	1.33	0.042 *	§
Angina	0	0	4	2.67	3	2.00	0.152	
IHD	15	10.00	16	10.67	10	6.67	0.435	
Stroke	18	12.00	9	6.00	7	4.67	0.038 *	#, §
AF	6	4.00	5	3.33	3	2.00	0.597	
HF	5	3.33	3	2.00	4	2.67	0.773	
**Comorbidities**								
HTN	22	14.67	14	9.33	21	14.00	0.318	
DM	19	12.67	19	12.67	18	12.00	0.980	
Hyperlipidemia	5	3.33	4	2.67	6	4.00	0.813	
COPD	9	6.00	8	5.33	10	6.67	0.889	
Cancer	11	7.33	10	6.67	13	8.67	0.800	
CKD	26	17.33	22	14.67	10	6.67	0.016 *	§, †
**Medications**								
β-blocker	13	8.67	15	10.00	15	10.00	0.902	
CCB	10	6.67	13	8.67	11	7.33	0.800	
ACEI	11	7.33	9	6.00	10	6.67	0.898	
ARB	8	5.33	8	5.33	9	6.00	0.959	
Diuretic	7	4.67	10	6.67	8	5.33	0.743	
Statin	8	5.33	7	4.67	9	6.00	0.876	
Steroid	4	2.67	3	2.00	4	2.67	0.911	

CV = cardiovascular, CAD = coronary artery disease, AMI = acute myocardial infarction, IHD = ischemic heart disease, AF = atrial fibrillation, HF = heart failure, HTN = hypertension, DM = diabetes mellitus, COPD = chronic obstructive pulmonary disease, CKD = chronic kidney disease, CCB = calcium channel blocker, ACEIs = angiotensin converting enzyme inhibitors, ARBs = angiotensin receptor blockers

p: Chi-square/Fisher’s exact test for categorical variables and one-way ANOVA for continuous variables

Post hoc test: Chi-square/Fisher’s exact test for categorical variables and Bonferroni for continuous variables

^#^ = significant between heat stroke and other heat-related illnesses,

^§^ = significant between heat stroke and non-heat-related illnesses,

^†^ = significant between other heat illnesses and nonheat illnesses

*p < 0.05,

**p < 0.01,

***p < 0.001

**Table 3:** Factors associated with cardiovascular diseases according to Cox regression in patients with heat stroke, other heat-related illnesses and Non-heat-ielated illnesses.

Heat-related illness	Heat stroke *vs*. Other heat-related illnesses *(reference)*		Heat stroke *vs*. Non-heat-related illnesses *(reference)*	
Events	Adjusted HR (95% CI)	p	Adjusted HR (95% CI)	p
**CV events** (overall)	1.485 (1.019–2.367)	0.030 *	3.899 (2.275–6.942)	< 0.001 ***
**CAD**	1.348 (0.685–2.674)	0.376	3.178 (1.537–6.972)	0.001 **
AMI	7.426 (1.194–45.785)	0.009 **	2.711 (1.849–13.001)	< 0.001***
Angina	-	-	-	-
IHD	1.120 (0.901–2.345)	0.138	3.124 (1.266–7.973)	0.003 **
Stroke	2.399 (1.275–5.899)	< 0.001 ***	5.498 (1.306–18.204)	< 0.001 ***
AF	1.085 (0.301–4.572)	0.899	14.978 (1.785–129.782)	< 0.001 ***
HF	1.426 (0.199–11.046)	0.726	26.601 (0.803–651.989)	0.064

CV = cardiovascular, CAD = coronary artery disease, AMI = acute myocardial infarction, IHD = ischemic heart disease, AF = atrial fibrillation, HF = heart failure

Adjusted HR = adjusted hazard ratio: adjusted for sex, age, comorbidities, annual medical visits and medications

CI = confidence interval

*p < 0.05,

**p < 0.01,

***p < 0.001

## Discussion

We have demonstrated an association between prior HS and an increased incidence of CVDs, specifically AMI and AIS, compared with those of age-, sex- and comorbidity-matched patients with other HRIs and controls in this population-based study. We speculate that the increase in CVDs may be due to HS itself revealing individuals susceptible to CVDs as a long-term sequela of HS.

Although the evidence suggests adverse impacts of heat waves on human health in many regions [13], morbidity and mortality related to heat conditions are reduced by appropriate preventions and treatments. However, the growing incidence of HRIs warrants early recognition of clinical risks in HRI patients with other CVDs. Pre-existing illness, a high BMI and CVDs can predispose individuals to HS [7]. Common clinical findings in patients with HS include multiple organ failure, rhabdomyolysis and systemic inflammation [6]. Patients with HS frequently have decreased diastolic blood pressure and oxygen saturation, an increased shock index and a reduced consciousness level [14]. In addition to heat cytotoxicity, recent clinical and experimental evidence suggests a complex interplay between coagulation and systemic inflammatory response syndrome, which results in damage to the gut and other organs [7]. The findings that HS could increase the risk of CVDs and IHDs were consistent with those of two previous studies of military personnel with severe HRIs and underground workers exposed to heat, which were associated with increased CVD-related mortality. In the Total Army Injury and Health Outcome Database, patients hospitalized due to severe HRIs were associated with increased CVD and IHD-related mortality [11]. Mortality from IHDs was higher for those workers with heat exposure [15]. Although we minimized the effects of comorbidities by matching between groups prior to the comparisons, we could not exclude the possibility of HS itself revealing patients who were susceptible to CVDs, such as those with metabolic syndrome and a high BMI. HS is frequently complicated with AKI. Up to 90.9% of patients suffer from AKI, of whom 16.7% receive acute dialysis [16]. AKI in survivors of critical illness predicts a worse frailty status after discharge [17]. The presence of AKI is also strongly associated with the risk of CVDs and death in critically ill patients [18]. Our study also revealed that patients with HS had a higher incidence of CKD during the follow-up period. Patients with CKD had significantly higher morbidity and mortality due to CVDs [19,20].

Previous studies have indicated that critical illness increases the risk of CVDs. Half of patients with pneumonia retain high circulating inflammatory markers for a period, and patients hospitalized for pneumonia have an increased short-term and long-term risk of CVDs [21,22]. Several studies addressed the risk of AMI after AKI in different patient populations. Patients admitted to the ICU with AKI stages 2 to 3 were associated with an increased risk of AMI [23]. Declined renal function after percutaneous coronary intervention and during AMI are strongly related to long-term mortality, CKD and an increased risk of late CVDs [24–27].

We also found that prior HS was associated with an increased incidence of AIS in this study. Brain tissue is vulnerable to heat [28]. Almost all patients with HS experienced acute neurological disturbances, and up to 23.3% of the HS patients suffered long-term neurological sequelae [8]. The patterns of neurological deficits included motor dysfunction in 66.7% of the patients, cognitive impairment in 9.5% and both motor and cognitive impairment in 19%. Long-term cerebellar dysfunction was found in 71.4% of those HS patients [8]. A previous study indicated that AIS was also increased after a critical illness, such as sepsis [29]. Cerebrovascular disease and AIS are very common at all stages of CKD and most likely represent both shared risk factors and synergy among risk factors [30]. Approximately 14% to 16% of Chinese stroke patients had CKD at various stages [31]. CKD was associated with an increased risk of all-cause mortality and recurrent stroke independent of traditional vascular risk factors [31]. Renal dysfunction on admission for AIS is common and is associated with poor outcomes [20]. In addition, patients with HS have an increased incidence of AF, which is clearly a risk factor for AIS.

Basic experiments revealed that the heat shock protein 72 level increased with heat stress in humans [32]. Furthermore, proinflammatory responses and autoimmune reactions to heat shock proteins in the vessel wall can contribute to the initiation and perpetuation of atherosclerosis [33]. Although these studies have demonstrated a relationship between heat stress and cardiovascular events, the evidence is still limited.

### Limitations

Although we extensively adjusted our results by utilizing matching and multivariate logistic regression, our study still exhibited several limitations. The NHIRD registry could not provide detailed information regarding BMI, family histories, occupation, health-related lifestyle factors and laboratory results, which might be potential confounding factors in this study. In addition, the NHIRD registry could not provide detailed information regarding the HRI severity. The COPD incidence was used as a proxy variable for tobacco use to neutralize the potential confounding effect of this variable on our study design [34]. In this study, HRIs were identified using the NHIRD registry. However, exertional and nonexertional HRIs cannot be distinguished using ICD-9 codes but have different etiologies and prevalence rates. Young, fit individuals may experience more exertional HS, and elders who have comorbidities may account for more nonexertional HS cases, which will be a confounder for the subsequent CVD results.

## Conclusion

Prior HS was associated with an increased incidence of CVDs, particularly AMI and AIS, and an increased incidence of CKD.

## Supporting information

S1 Fig Histogram of the ages at the time of HS and HRI onset. (TIF)
