# Supplementary figures and images for: The association between heat stroke and subsequent cardiovascular diseases
Source: PLoS One. 2019 Feb 13;14(2):e0211386. doi: 10.1371/journal.pone.0211386 (PMC6373898; doi:10.1371/journal.pone.0211386)

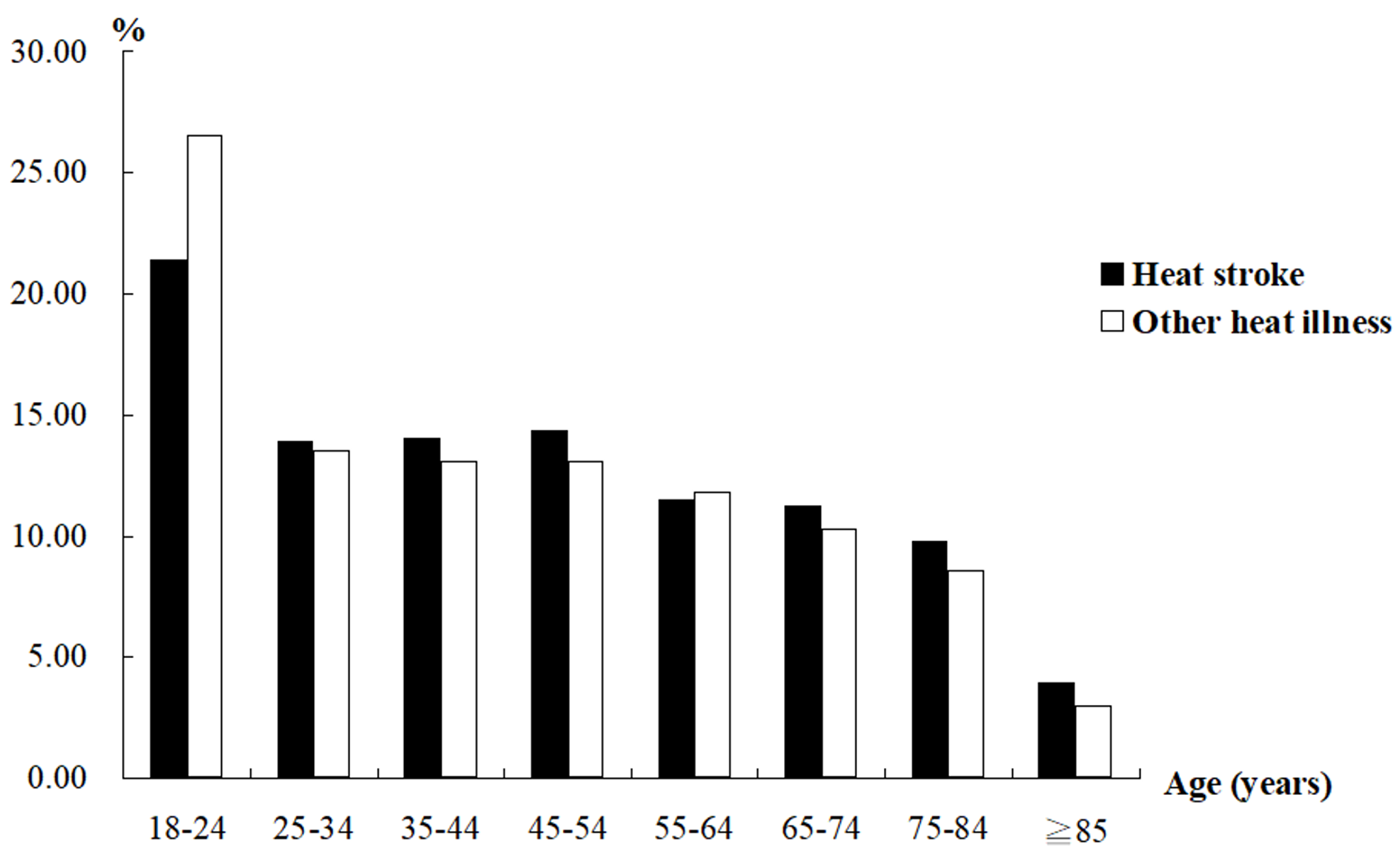

Supplement: S1 Fig — (TIF) [file pone.0211386.s001.tif]
